# Efficacy of adjuvant chemotherapy on overall survival in patients with lymph node‐positive esophageal squamous cell carcinoma: Is oral chemotherapy promising?

**DOI:** 10.1002/cam4.5264

**Published:** 2022-09-22

**Authors:** Shuogui Fang, Jian Zhong, Zihang Mai, Tong Li, Xiuying Xie, Jianhua Fu

**Affiliations:** ^1^ Department of Thoracic Oncology Sun Yat‐sen University Cancer Center Guangzhou China; ^2^ State Key Laboratory of Oncology in South China Collaborative Innovation Center for Cancer Medicine Guangzhou China; ^3^ Guangdong Esophageal Cancer Institute Guangzhou China; ^4^ Department of Cancer Prevention Research Sun Yat‐sen University Cancer Center Guangzhou China

**Keywords:** adjuvant chemotherapy, esophageal squamous cell carcinoma, S‐1, survival

## Abstract

**Background:**

The role of adjuvant chemotherapy in patients with pathological lymph node‐positive (pN+) resectable esophageal squamous cell carcinoma (ESCC) remains unclear. We aimed to explore whether adjuvant chemotherapy could improve the overall survival (OS) of patients with pN+ ESCC and whether oral chemotherapy could be used as an alternative to intravenous chemotherapy.

**Methods:**

The patients were divided into two groups: a surgery plus chemotherapy group (S + CT group, 400 patients) and a surgery alone group (S group, 582 patients). Propensity score matching (PSM) was used to create patient groups that were balanced across several covariates (*n* = 331 in each group). The survival rates of patients receiving oral chemotherapy (69 patients with S‐1 and 68 patients with tegafur tablets) and intravenous chemotherapy (263 patients) were compared using the Kaplan–Meier method.

**Results:**

In the overall study cohort, the 3‐year OS was significantly higher in the S + CT group than in the S group (66.3% vs. 49.9%, *p* < 0.001). These data were confirmed in the matched groups (3‐year OS, 72.9% vs. 62.0%, *p* < 0.001). Multivariate Cox regression analysis in the matched samples showed that adjuvant chemotherapy was an independent prognostic factor for ESCC (HR: 0.62, 95% CI: 0.50–0.76, *p* < 0.001). Patients who received oral chemotherapy had a similar OS as patients who received intravenous chemotherapy.

**Conclusions:**

Adjuvant chemotherapy could significantly improve the OS of patients with pN+ ESCC, and oral chemotherapy drugs might be a better option because of their similar efficacy but fewer side effects than intravenous chemotherapy. This conclusion warrants further study in prospective, randomized controlled trials.

## 1 INTRODUCTION

Surgery has been the primary treatment for patients with local advanced esophageal cancer; however, the prognosis for patients who receive surgery alone is very poor, with a 5‐year overall survival rate of only 25%–49.1%, mainly due to the high recurrence and distant metastasis rate.^1^, ^2^, ^3^, ^4^ Therefore, it is necessary to explore treatment options in addition to surgery. Although the role of preoperative treatment in ESCC is relatively clear, there are still many patients who do not receive neoadjuvant therapy, which sometimes leads to poor prognosis, especially for patients with positive pathological lymph nodes. Therefore, postoperative adjuvant therapy, such as adjuvant chemotherapy, might be a solution for the above problems. Although adjuvant chemotherapy has been recommended for postoperative treatment in patients with lymph node‐positive esophageal adenocarcinoma after R0 resection,^5^ further studies are needed to clarify its role in ESCC.

Many studies have explored the effect of adjuvant chemotherapy on the prognosis of ESCC, but there is no consensus to date.^6^, ^7^, ^8^, ^9^ Some research results have shown that adjuvant chemotherapy can improve the disease‐free survival (DFS) of patients with ESCC but has no significant effect on OS.^8^, ^9^, ^10^ However, other studies concluded that adjuvant chemotherapy could not only improve DFS but also improve OS, especially in patients with pN+ ESCC.^11^, ^12^, ^13^ Because patients with positive lymph nodes have a worse survival time than those with negative lymph nodes, it is important to identify suitable adjuvant treatments for these patients.^14^ Therefore, the first purpose of this study was to explore whether adjuvant chemotherapy can improve OS in patients with positive lymph nodes.

Some patients, especially elderly patients, cannot tolerate postoperative adjuvant chemotherapy due to its side effects. Therefore, it would be very beneficial to identify a chemotherapy regimen that has fewer side effects and is more convenient for elderly patients. S‐1 is an oral anticancer drug derived from fluorouracil and has the advantages of convenient oral administration and slight side effects,^15^ while tegafur is the main component of S‐1. However, whether S‐1 and tegafur tablets have similar effects to intravenous chemotherapy in the adjuvant setting is still unclear. The study also aims to explore whether these oral chemotherapy drugs can be used as an alternative to intravenous chemotherapy.

## 2 MATERIALS AND METHODS

### 2.1 Patient selection

This single‐center retrospective study was conducted based on a database of inpatients from the Sun Yat‐Sen University Cancer Center between January 2011 and December 2020. The inclusion criteria were as follows: (1) Patients who were diagnosed with ESCC pathologically or histologically; (2) patients who underwent surgery alone without any perioperative antitumor treatment and patients who had adjuvant chemotherapy after esophagectomy; (3) patients with pathological lymph node‐positive ESCC; (4) patients who received R0 esophagectomy. The exclusion criteria were as follows: (1) Patients who had a history of other cancers and (2) patients who died within 30 days of esophagectomy or in the hospital. Tumor pathological staging for all patients was based on the eighth edition of the American Joint Committee on Cancer (AJCC) tumor–node–metastasis (TNM) classification. This study was approved by the local ethical committee of Sun Yat‐sen University Cancer Center. The need for written informed consent was waived due to the retrospective nature of the study.

### 2.2 Follow‐up

Patients were followed up every 3–6 months for the first 2 years after surgery, every 6 months for the third to fifth years, and once a year after 5 years. The routine follow‐up workup included physical examination, enhanced CT scan of the chest and abdomen, and ultrasound examination of the neck. OS was set as the primary endpoint, which was defined as the duration from the date of surgery to the date of death from any cause.

### 2.3 Statistical analysis

Statistical analysis was performed using SPSS 13.0 software (SPSS Inc.) and R language (Version 3.6.1). The chi‐square test and Student's *t* test were used to compare baseline characteristics between the two groups. PSM was performed using the R language with the variables age, sex, tumor location, and the American Society of Anesthesiologists (ASA) score, which were significantly different between the two groups, and the caliper width was 0.02. The median survival time of patients was estimated by the Kaplan–Meier method and compared by the log‐rank test. Multivariate analysis was performed to investigate the prognostic factors by the Cox proportional hazard regression model, and the hazard ratios (HRs) and 95% confidence intervals (CIs) were estimated. Factors with a *p* value <0.1 in the univariate analysis were included in the Cox regression model. All statistical tests were two‐sided, and a *p* value <0.05 was considered statistically significant.

## 3 RESULTS

### 3.1 Baseline characteristics of the patients

**FIGURE 1:**
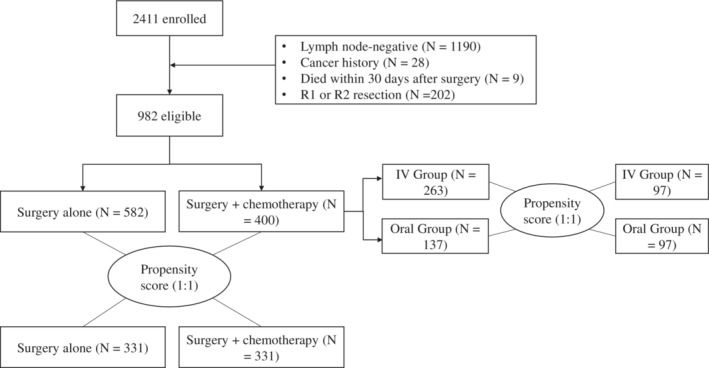
CONSORT flow diagram

A total of 982 patients were initially selected. In the preliminary analysis, age, sex, tumor location, and ASA score were significantly different between the S + CT group and S group (see Table 1). To balance these factors, propensity score matching (PSM) was adopted to match each patient undergoing adjuvant chemotherapy with one patient undergoing surgery alone. From this stratification process, a total of 662 patients were finally selected, with 331 patients in each group (Figure 1). In the matched cohort, the median age was 60 years (range 35–79 years), and 544 (82.2%) patients were male. The McKeown procedure was the most common surgical method, with 323 patients (48.8%), followed by the Sweet procedure with 223 patients (33.7%) and the Ivor Lewis procedure with 116 patients (17.5%). There was no statistically significant difference in baseline characteristics between the two groups after matching, including age, sex, tumor stage, tumor location, surgical approach, degree of tumor differentiation, ASA score, number of lymph nodes examined, anastomotic leakage rate, and wound infection rate (Table 1).

**TABLE 1:** Baseline characteristics of the study population before and after propensity score matching

Variable	Overall (%) *N* = 982	Before matching			After matching		
		S group (cases, %)	S + CT group (cases, %)	*p* Value	S group (cases, %)	S + CT group (cases, %)	*p* Value
Gender				0.002			0.761
Male	782 (79.6)	444 (76.3)	338 (84.5)		274 (82.8)	270 (81.6)	
Female	200 (20.4)	138 (23.7)	62 (15.5)		57 (17.2)	61 (18.4)	
Age (year)				<0.001			0.482
<60	412 (42.0)	215 (36.9)	197 (49.2)		153 (46.2)	143 (43.2)	
≥60	570 (58.0)	367 (63.1)	203 (50.7)		178 (53.8)	188 (56.8)	
Pathological T stage				0.545			0.823
pT1	82 (8.4)	46 (7.9)	36 (9.0)		28 (8.5)	28 (8.5)	
pT2	143 (14.6)	92 (15.8)	51 (12.8)		42 (12.7)	50 (15.1)	
pT3	733 (74.6)	429 (73.7)	304 (76.0)		252 (76.1)	243 (73.4)	
pT4	24 (2.4)	15 (2.6)	9 (2.2)		9 (2.7)	10 (3.0)	
Pathological *N* stage				0.123			0.758
pN1	577 (58.8)	356 (61.2)	221 (55.2)		182 (55.0)	191 (57.7)	
pN2	307 (31.3)	175 (30.1)	132 (33.0)		111 (33.5)	106 (32.0)	
pN3	98 (10.0)	51 (8.8)	47 (11.8)		38 (11.5)	34 (10.3)	
Pathological stage				0.748			0.768
IIB	57 (5.8)	36 (6.2)	21 (5.2)		16 (4.8)	22 (6.6)	
IIIA	119 (12.1)	70 (12.0)	49 (12.2)		40 (12.1)	42 (12.7)	
IIIB	697 (71.0)	416 (71.5)	281 (70.2)		235 (71.0)	227 (68.6)	
IVA	109 (11.1)	60 (10.3)	49 (12.2)		40 (12.1)	40 (12.1)	
Tumor location				0.014			0.924
Upper	73 (7.4)	51 (8.8)	22 (5.5)		22 (6.6)	23 (6.9)	
Middle	520 (53.0)	320 (55.0)	200 (50.0)		163 (49.2)	167 (50.5)	
Lower	389 (39.6)	211 (36.3)	178 (44.5)		146 (44.1)	141 (42.6)	
Operation approach				0.669			0.504
Ivor Lewis	172 (17.5)	107 (18.4)	65 (16.2)		55 (16.6)	61 (18.4)	
McKeown	470 (47.9)	274 (47.1)	196 (49.0)		169 (51.1)	154 (46.5)	
Sweet	340 (34.6)	201 (34.5)	139 (34.8)		107 (32.3)	116 (35.0)	
Differentiated degree				0.761			0.691
Poorly	370 (37.7)	214 (36.8)	156 (39.0)		130 (39.3)	139 (42.0)	
Moderately	484 (49.3)	290 (49.8)	194 (48.5)		161 (48.6)	150 (45.3)	
Highly	128 (13.0)	78 (13.4)	50 (12.5)		40 (12.1)	42 (12.7)	
Numbers of lymph node examined				0.421			0.411
<15	123 (12.5)	77 (13.2)	46 (11.5)		45 (13.6)	38 (11.5)	
≥15	859 (87.5)	505 (86.8)	354 (88.5)		286 (86.4)	293 (88.5)	
ASA score				0.040			0.852
1–2	932 (94.9)	546 (93.8)	386 (96.5)		315 (95.2)	317 (95.8)	
3–4	50 (5.1)	36 (6.2)	14 (3.5)		16 (4.8)	14 (4.2)	
Anastomotic leakage				0.134			0.360
Yes	119 (12.1)	63 (10.8)	56 (14.0)		40 (12.1)	48 (14.5)	
No	863 (8709)	519 (89.2)	344 (86.0)		291 (87.9)	283 (85.5)	
Wound infection				1.000			1.000
Yes	9 (0.9)	5 (0.9)	4 (1.0)		3 (0.9)	3 (0.9)	
No	973 (99.1)	577 (99.1)	396 (99.0)		328 (99.1)	328 (99.1)	

Abbreviation: ASA, American Society of Anesthesiologists.

### 3.2 Treatment

All patients underwent a transthoracic total esophagectomy and end‐to‐side esophagogastric anastomosis through left or right thoracotomy. Two‐field lymphadenectomy with total mediastinal lymph node dissection was performed, and dissection of the left and right recurrent laryngeal nerve nodes was mandatory in surgery through the right thoracic approach according to our previous report.^16^ Among the 2411 patients initially included, nine patients died within 30 days after surgery, and 202 patients (8.4%) underwent R1/R2 resection and were excluded from the analysis. For the 982 patients who were finally included, 345 patients (35.1%) underwent minimally invasive surgery, while the remaining 637 (64.9%) underwent conventional open surgery. Patients who received esophagectomy were routinely sent to the ICU for monitoring after surgery in our hospital, but none of the above 982 patients had serious complications, and they all returned to the general ward after 1 day of monitoring in the ICU.

Among 400 patients who received postoperative chemotherapy, 41.8% (167 of 400) of patients received docetaxel‐based regimens, 24.0% (96 of 400) received paclitaxel‐based regimens, 17.2% (69 of 400) received S‐1 plus celecoxib regimens, and the remaining 17.0% (68 of 400) received tegafur tablet therapy. The drugs combined with docetaxel or paclitaxel included cisplatin, nedaplatin, oxaliplatin, or carboplatin. The details of these regimens are summarized in Table S1. The median interval between the date of surgery and the date of the first postoperative cycle chemotherapy was 5.61 weeks, ranging from 0.72 to 13.25 weeks. Since there is no unified opinion on postoperative adjuvant chemotherapy, doctors and the patients decided whether to carry out chemotherapy.

### 3.3 Survival analysis

**FIGURE 2:**
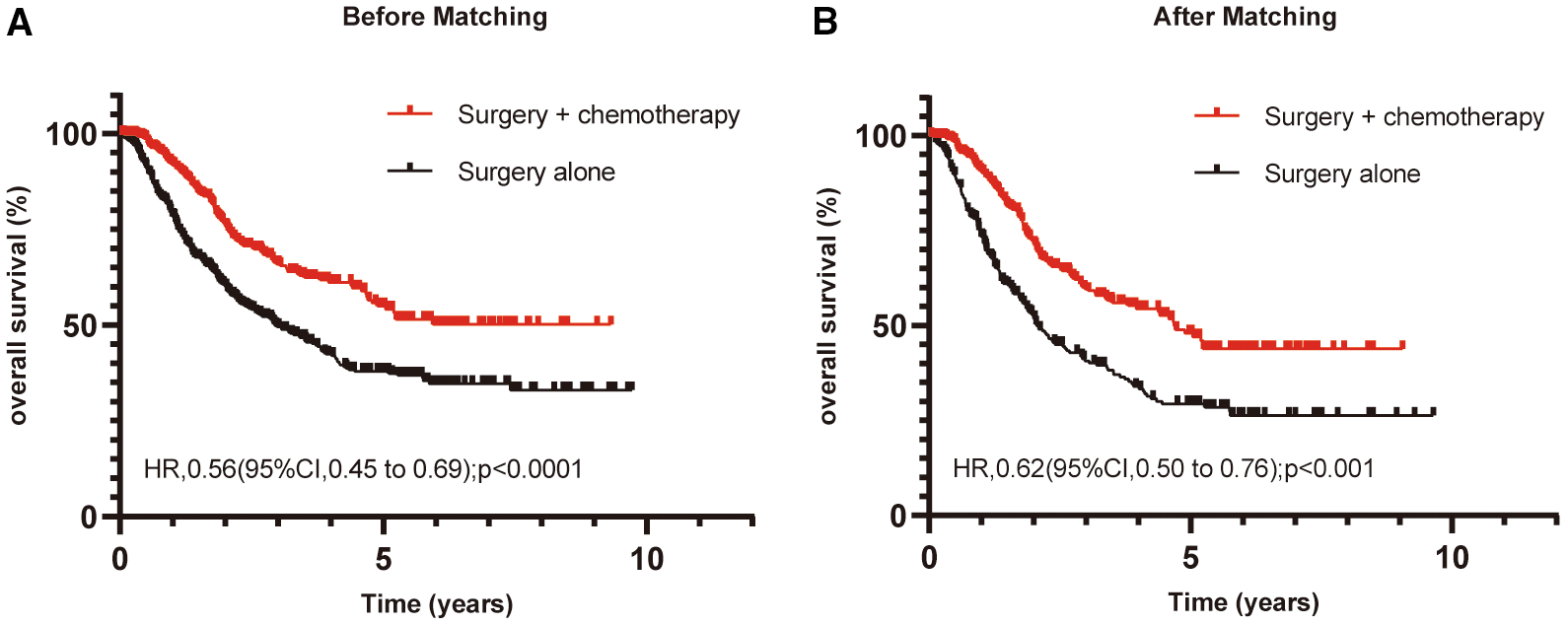
Kaplan–Meier curves for OS in unmatched (A) and matched (B) ESCC patients classified into surgery plus chemotherapy and surgery alone groups. OS, overall survival.

The median follow‐up was 36.6 months across the whole study population (interquartile range [IQR], 19.5–68.9 months). In the overall study cohort before PSM, the 3‐year OS rate in the S + CT group was significantly higher than that in the S group (66.3% vs. 49.9%, *p* < 0.001) (Figure 2A). This finding was confirmed in the matched samples. In the S + CT group, the 3‐year OS rate was 72.9%, which was significantly higher than that in the S group (62.0%, *p* < 0.001; Figure 2B). The median OS was 50.2 months (95% CI: 40.9–59.4 months) for the S group, which was not reached in the S + CT group. To further clarify which lymph node metastatic status is effective for adjuvant chemotherapy, we performed a subgroup analysis and found that in the original case, adjuvant chemotherapy was effective in patients with pN1, pN2, and pN3. In the balanced cohort, adjuvant chemotherapy was effective in patients with pN1 and pN2, except for patients with pN3, which may account for the limited number of patients with pN3 (Figure S1).

To explore the factors that may influence the OS of patients, univariate analysis was conducted by the log‐rank test in the matched samples. We found that age, pathological T stage, pathological *N* stage, and adjuvant chemotherapy were associated with the survival of patients. Further multivariate Cox regression was performed by adjusting the above associated factors, and adjuvant chemotherapy was found to be an independent prognostic factor of OS in patients with pN+ ESCC (HR: 0.62, 95% CI: 0.50–0.76, *p* < 0.001; see Table 2).

**TABLE 2:** Univariate analysis and multivariate analysis of prognostic factors for OS

Variable	Univariate analysis		Multivariate analysis	
	HR (95% CI)	*p* value	HR (95% CI)	*p* value
Chemotherapy (Yes vs. No)	0.62 (0.50–0.76)	<0.001	0.62 (0.50–0.76)	<0.001
Age (≥60 vs. <60)	1.75 (1.42–2.15)	<0.001	1.74 (1.41–2.16)	<0.001
Sex (male vs. female)	1.53 (1.16–2.01)	0.002	1.35 (1.02–1.78)	0.038
Pathological T stage				
pT2 versus pT1	1.64 (0.93–2.91)	0.088	1.63 (0.92–2.89)	0.094
pT3 versus pT1	3.08 (1.86–5.09)	<0.001	2.69 (1.62–4.46)	<0.001
pT4 versus pT1	5.53 (2.61–11.69)	<0.001	5.60 (2.62–11.98)	<0.001
Pathological *N* stage				
pN2 versus pN1	1.58 (1.27–1.98)	<0.001	1.66 (1.32–2.08)	<0.001
pN3 versus pN1	2.96 (2.16–4.05)	<0.001	2.89 (2.09–3. 99)	<0.001
Pathological stage				
IIB versus IVA	1.46 (0.70–3.05)	0.312		
IIIA versus IVA	3.19 (1.64–6.20)	0.001		
IIIB versus IVA	6.75 (3.33–13.70)	<0.001		
Differentiation degree				
Highly versus poorly	0.96 (0.69–1.35)	0.825		
Moderately versus poorly	1.00 (0.80–1.25)	0.986		
Tumor location				
Distal third versus proximal third	1.12 (0.74–1.69)	0.588		
Middle third versus proximal third	1.09 (0.73–1.63)	0.683		
Operation approach				
Ivor Lewis versus sweet	0.89 (0.68–1.17)	0.409	0.78 (0.59–1. 04)	0.086
McKeown versus sweet	0.71 (0.56–0.88)	0.002	0.67 (0.53–0. 84)	0.001

### 3.4 Subgroup analysis of chemotherapy regimen stratified by administration route

**FIGURE 3:**
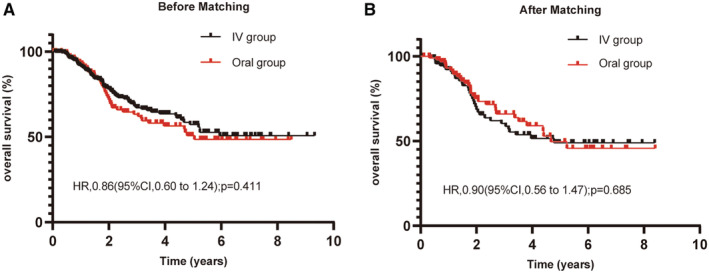
Kaplan–Meier curves for OS in unmatched (A) and matched (B) ESCC patients classified into the oral group and IV group. IV, intravenous chemotherapy.

In the S + CT group, we noticed that the chemotherapy regimen could be divided into two categories: one was S‐1 and tegafur tablets that were taken orally (oral group), and the other was administered intravenously (IV group), including docetaxel‐based regimens and paclitaxel‐based regimens. We compared the toxicity of these two chemotherapy regimens and found that oral chemotherapy was less toxic than intravenous chemotherapy (Table S2). Since S‐1 and tegafur tablets also have the advantages of convenient oral administration, we continued to explore whether this regimen could be an alternative to intravenous chemotherapy. In the overall population, there were 137 patients in the oral group and 263 patients in the IV group. We found that the oral group had a 3‐year OS similar to that of the IV group (62.7% vs. 67.9%, *p* = 0.411; Figure 3A). After PSM, there was still no significant difference in the 3‐year OS between the above two groups (60.3% vs. 65.9%, *p* = 0.685; Figure 3B). The baseline characteristics of these two groups are summarized in Table 3.

**TABLE 3:** Baseline characteristics of patients who received two different kinds of chemotherapy

	No. patients	IV group (cases, %)	Oral group (cases, %)	*p*
Regimens	194	Docetaxel‐based OR Paclitaxel‐based (*N* = 97)	S‐1 OR Tegafur tablets (*N* = 97)	
Gender				
Male	172 (88.7)	85 (87.6)	87 (89.7)	0.840
Female	22 (11.3)	12 (12.4)	10 (10.3)	
Age (year)				1
<60	79 (40.7)	39 (40.2)	40 (41.2)	
≥60	115 (59.3)	58 (59.8)	57 (58.8)	
Pathological T stage				0.170
pT1‐2	38 (19.6)	13 (13.4)	25 (25.8)	
pT3‐4	156 (80.4)	84 (86.6)	72 (74.2)	
Pathological *N* stage				0.386
pN1	107 (55.2)	50 (51.5)	57 (58.8)	
pN2‐3	87 (44.8)	47 (48.5)	40 (41.2)	
Pathological stage				0.176
IIB‐IIIA	32 (16.5)	12 (12.4)	20 (20.6)	
IIIB‐IVA	162 (83.5)	85 (87.6)	77 (79.4)	
Tumor location				0.838
Upper	5 (2.6)	2 (2.1)	3 (3.1)	
Middle	112 (57.7)	55 (56.7)	57 (58.8)	
Lower	77 (39.7)	40 (41.2)	37 (38.1)	
Operation approach				0.093
Ivor Lewis	32 (16.5)	13 (13.4)	19 (19.6)	
McKeown	96 (49.5)	44 (45.4)	52 (53.6)	
Sweet	66 (34.0)	40 (41.2)	26 (26.8)	
Differentiated degree				0.304
Poorly	73 (37.6)	39 (40.2)	34 (35.1)	
Moderately	96 (49.5)	43 (44.3)	53 (54.6)	
Highly	25 (12.9)	15 (15.5)	10 (10.3)	

## 4 DISCUSSION

In the current study, adjuvant chemotherapy after radical resection was associated with significantly improved 3‐year OS rates in pN+ ESCC. The survival benefits from postoperative chemotherapy were maintained in the propensity score‐matched analysis. Oral chemotherapy had a similar effect to intravenous chemotherapy on the OS of patients with ESCC, with the advantage of oral administration and fewer side effects. Thus, oral chemotherapy can be used as an alternative to intravenous chemotherapy.

Although neoadjuvant chemoradiotherapy has been shown to significantly improve the prognosis of locally advanced ESCC, a considerable number of patients still do not receive neoadjuvant therapy. In our center, the proportion of patients receiving neoadjuvant therapy is only approximately 30%. The reasons why patients did not receive neoadjuvant chemoradiotherapy may be as follows. First, in China, patients with ESCC generally have poor economic conditions and cannot afford the cost of chemoradiotherapy. Second, patients were worried about the side effects of chemoradiotherapy and hoped to have radical resection as soon as possible. Third, some patients' clinical *N* status could not be determined.

For those patients with positive pathological lymph nodes, postoperative adjuvant therapy, such as postoperative adjuvant chemotherapy, will be an important option. However, the role of adjuvant chemotherapy for patients with resected ESCC remains unclear. Some research has shown that adjuvant chemotherapy can improve the disease‐free survival (DFS) of patients with ESCC, but it has no significant effect on OS. In 2003, Ando et al. conducted a large‐scale multicenter prospective study (JCOG9204). A total of 242 patients with esophageal squamous cell carcinoma were enrolled. Among them, 122 patients received surgery alone, and 120 received adjuvant chemotherapy after surgery, and the annual progression‐free survival rates were 45% and 55%, respectively (*p* = 0.037), suggesting that postoperative chemotherapy has a preventive effect on postoperative recurrence of esophageal cancer. The 5‐year OS rates of the two groups of patients were 52% and 61%, respectively. Although the difference was not statistically significant (*p* = 0.13), there was a trend indicating improvement.^8^ Recently, a large‐scale, multicenter, real‐world study also found that adjuvant chemotherapy showed the best overall survival in pN+ ESCC, although the finding was not statistically significant.^17^

Some studies have reported that adjuvant chemotherapy could significantly improve both DFS and OS. In a prospective phase II trial, the authors concluded that biweekly adjuvant PTX and CDDP (4–6 cycles of PTX 150 mg/m^2^ administered intravenously on day 1 followed by CDDP 50 mg/m^2^ on day 2 every 14 days) might improve 3‐year DFS and OS in lymph node‐positive, curatively resected thoracic ESCC patients (DFS: 56.3% vs. 34.6%, *p* = 0.006; OS: 55.0% vs. 37.5%, *p* = 0.013).^11^ A meta‐analysis showed that adjuvant chemotherapy did not significantly prolong 3‐year OS (risk ratio [RR] = 0.89, 95% CI, 0.72–1.09; *p* = 0.25), but it significantly improved the 3‐year OS of patients with stage III–IV disease in a further subgroup analysis (RR = 0.43, 95% CI, 0.31–0.61; *p* = 0.00001), suggesting that patients with positive lymph nodes may benefit more from adjuvant chemotherapy, which is similar to our results.^10^ Another meta‐analysis also concluded that postoperative chemotherapy could improve OS (HR 0.78, 95% CI 0.66–0.91; *p* = 0.002) and DFS (HR 0.72, 95% CI 0.6–0.86; *p* < 0.001).^12^

In summary, most studies believe that adjuvant chemotherapy could improve the DFS of ESCC, but there are different conclusions on whether it can prolong the OS of patients. The conflicting results of these studies might be due to differences in tolerance to chemotherapy, the timing of chemotherapy initiation, and the number of chemotherapy cycles. However, the above‐mentioned studies suggest that adjuvant chemotherapy prolongs the overall survival of patients with pathological lymph nodes. Therefore, this study aimed to explore whether adjuvant chemotherapy can improve the OS of patients with pN+ ESCC. We found that adjuvant chemotherapy could prolong the 3‐year OS by 16.4% (from 49.9% to 66.3%, *p* < 0.001), and it was an independent prognostic factor for the OS of patients with pN + ESCC. A recent study reported a similar result to ours, but they did not investigate whether oral chemotherapeutics could be substituted for intravenous chemotherapeutics to reduce adverse effects.^18^ The reasons for our positive results might be as follows. First, in the intravenous chemotherapy regimen, we used a two‐drug combination regimen including taxane, while most other studies used a 5‐fluorouracil combination regimen.^8^, ^9^ Studies have shown that taxane is more beneficial for the adjuvant treatment of esophageal squamous cell carcinoma.^19^ Second, less toxic platinums, such as nedaplatin, were used in intravenous chemotherapy. Third, the median number of cycles of intravenous chemotherapy in this study was 4, while that in other studies was mostly 2–3.^8^, ^9^ Finally, our chemotherapy regimen also included less toxic oral chemotherapeutics, S‐1, and tegafur tablets.

Although widely used in the treatment of many common cancers, the mainstream use of S‐1 is combination chemotherapy with other chemotherapeutic drugs.^20^, ^21^, ^22^, ^23^ However, S‐1 monotherapy is becoming an increasing treatment option and has shown good efficacy.^24^, ^25^, ^26^ Katsuhiko Uesaka et al. reported that adjuvant monotherapy with S‐1 could significantly improve the overall survival and relapse‐free survival among Japanese patients after pancreatic cancer resection, and patients were well tolerated.^15^ A phase II randomized trial conducted by Okamoto, T et al. found that in patients with pathological stage II/IIIA non‐small cell lung cancer, the 2‐year DFS rates were 52% (95% CI: 0.40–0.63) and 61% (95% CI: 0.48–0.70) for the S‐1 monotherapy group and S‐1 plus cisplatin group, respectively. Neither DFS nor OS differed significantly between the two arms (log‐rank test: *p* = 0.1695 for DFS and *p* = 0.8684 for OS, respectively).^27^ In this retrospective study, we found that S‐1 and tegafur tablet chemotherapy have similar effects to intravenous chemotherapy, but as mentioned earlier, they have the advantages of convenient oral administration and fewer side effects, so oral chemotherapy may be a better choice for patients with pN+ ESCC, especially for elderly individuals.

In addition to cytotoxic drugs, immune checkpoint blockades are also promising drugs. In recent years, immunotherapy has revolutionized the treatment of cancer, and the study of immunotherapy for adjuvant therapy of esophageal cancer has aroused extensive interest from researchers. Recently, the results of a study called Checkmate 577 showed that postoperative adjuvant nivolumab treatment can significantly improve disease‐free survival in patients with resected (R0) stage II or III esophageal or gastroesophageal junction cancer who had received neoadjuvant chemoradiotherapy and had residual pathological disease, but it remains unclear whether this treatment confers benefits for OS. In addition, immune checkpoint inhibitors for postoperative adjuvant therapy in patients with esophageal cancer who did not receive neoadjuvant chemoradiotherapy have not yet been examined; we look forward to research in this area. Because immunotherapy has fewer side effects, if postoperative adjuvant immunotherapy is confirmed to improve the overall survival of patients with esophageal cancer in the future, then postoperative adjuvant immunotherapy will hopefully become the standard treatment for esophageal cancer.

This study was not free from limitations. First, the bias inherent in the nature of retrospective investigations has not been completely avoided, such as selection bias and recall bias. Second, although PSM was used to balance important variables, some undocumented potential confounding factors, such as performance status and comorbidities, were not adjusted in the study. The third limitation was the small sample size used to compare the effect of oral chemotherapy versus intravenous chemotherapy on survival.

In conclusion, the study provided evidence that adjuvant chemotherapy significantly improved the OS of patients with pN+ ESCC and that oral chemotherapy contributed equally to intravenous chemotherapy. Moreover, as S‐1 and tegafur tablets can be prescribed in an outpatient clinic and taken orally at home, thereby improving patient adherence, orally administered adjuvant therapy may be a better choice for patients with pN+ ESCC.

## 5 CONCLUSION

Adjuvant chemotherapy can significantly improve the overall survival of patients with pN+ ESCC, and S‐1 or tegafur tablets may be a better choice of regimen. This conclusion warrants further studies in prospective, randomized controlled trials.

## AUTHOR CONTRIBUTIONS

**Shuo‐Gui Fang:** Data curation (equal); formal analysis (equal); methodology (equal); writing – original draft (equal). **Jian Zhong:** Data curation (equal); formal analysis (equal); methodology (equal). **zi‐Hang Mai:** Data curation (equal); writing – review and editing (equal). **Tong Li:** Formal analysis (supporting); methodology (supporting); writing – review and editing (supporting). **Xiuying Xie:** Data curation (supporting); writing – review and editing (supporting). **Jian‐Hua Fu:** Conceptualization (lead); funding acquisition (lead); resources (lead); supervision (lead); writing – review and editing (supporting).

## FUNDING INFORMATION

The data collection and editing of the English language in this present study were supported by the Health & Medical Collaborative Innovation Project of Guangzhou City, China (201803040018) and Guangdong Natural Science Foundation, China (2019A1515010715).

## Supporting information


Figure S1

Table S1

Table S2

## Data Availability

Not applicable.
