# Supplementary figures and images for: Efficacy of adjuvant chemotherapy on overall survival in patients with lymph node‐positive esophageal squamous cell carcinoma: Is oral chemotherapy promising?
Source: Cancer Med. 2022 Sep 22;12(4):4077–86. doi: 10.1002/cam4.5264 (PMC9972109; doi:10.1002/cam4.5264)

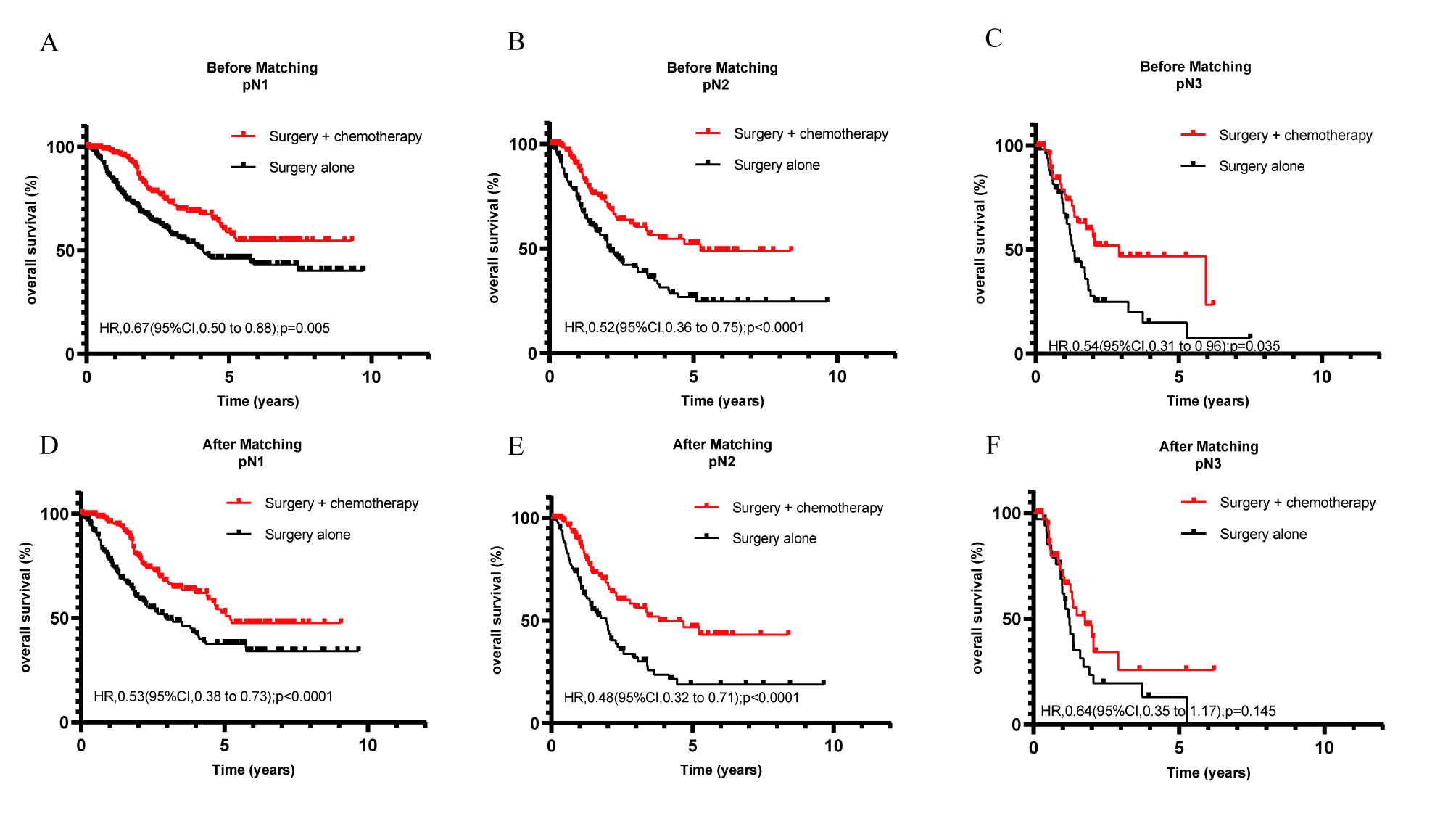

Supplement: Supplementary file 1 — Figure S1 [file CAM4-12-4077-s003.tif]
