# Supplementary material for: Efficacy of adjuvant chemotherapy on overall survival in patients with lymph node‐positive esophageal squamous cell carcinoma: Is oral chemotherapy promising?
Source: Cancer Med. 2022 Sep 22;12(4):4077–86. doi: 10.1002/cam4.5264 (PMC9972109; doi:10.1002/cam4.5264)
Supplement: Supplementary file 2 — Table S1 [file CAM4-12-4077-s001.docx]

Supplemental table 1. Detail of the chemotherapy regimens that 400 patients received

| Regimen | Usage and Dosage | Cycles  (median, range) | Patients(N) |
| --- | --- | --- | --- |
| DP | Docetaxel 55~80 mg/m^2^ IV d1 plus:  Nedaplatin 55~75 mg/m^2^ IV d1, N = 131;  OR Lobaplatin 20~30 mg/m^2^ IV d1, N = 28;  OR Cisplatin, 70~75 mg/m^2^ IV d1, N = 8.  Cycled every 3 weeks | 4(1-8) | 167 |
| TP | Paclitaxel 150~175 mg/m^2^ IV d1 plus:  Nedaplatin 80~100 mg/m^2^ IV d1, N = 51;  OR Carboplatin AUC 5 IV d1, N = 16;  OR Cisplatin, 70~75 mg/m^2^ IV d1, N = 15;  OR Lobaplatin 50 mg/m^2^ IV d1, N = 14;  Cycled every 3 weeks | 4(1-8) | 96 |
| S-1 | S-1 40-60 mg BID PO d1~14 plus  Celebrex 0.2mg QD PO d1~14.  Cycled every 3 weeks for up to 1 year | _ | 69 |
| Tegafur Tablets | 200 mg TID PO, 30~50g for one cycle | 2(1-7) | 68 |
| Total |  | 3(1-9) | 400 |
