# Supplementary material for: Efficacy of adjuvant chemotherapy on overall survival in patients with lymph node‐positive esophageal squamous cell carcinoma: Is oral chemotherapy promising?
Source: Cancer Med. 2022 Sep 22;12(4):4077–86. doi: 10.1002/cam4.5264 (PMC9972109; doi:10.1002/cam4.5264)
Supplement: Supplementary file 3 — Table S2 [file CAM4-12-4077-s002.docx]

Supplemental table 2 Adverse events in patients receiving chemotherapy by different routes of administration

|  | Routes of administration | | | | |  | |
| --- | --- | --- | --- | --- | --- | --- | --- |
|  | Intravenous  (n=263) | |  | Oral  (n=137) | |  |  |
| Adverse Event | Grade1-2 | Grade3-4 |  | Grade1-2 | Grade3-4 | *P* for Difference in Grade 1-2 | *P* for Difference in Grade 3-4 |
| Leukopenia | 18 (6.8) | 17 (6.5) |  | 3 (2.2) | 0 (0.0) | 0.048 | 0.002 |
| Neutropenia | 20 (7.6) | 10 (3.8) |  | 5 (3.6) | 0 (0.0) | 0.121 | 0.048 |
| Anemia | 45 (17.1) | 2 (0.8) |  | 2 (1.5) | 0 (0.0) | 0.000 | 0.548 |
| Thrombocytopenia | 6 (2.3) | 1 (0.4) |  | 0 (0.0) | 0 (0.0) | 0.075 | 1.000 |
| Hepatic dysfunction | 28 (10.6) | 15 (5.7) |  | 5 (3.6) | 0 (0.0) | 0.016 | 0.004 |
| Vomiting | 31 (11.8) | 16 (6.1) |  | 3 (2.2) | 0 (0.0) | 0.001 | 0.003 |
| Diarrhea | 23 (8.7) | 12 (4.6) |  | 2 (1.5) | 0 (0.0) | 0.004 | 0.011 |
| Anorexia | 41 (15.6) | 18 (6.8) |  | 9 (6.6) | 0 (0.0) | 0.010 | 0.002 |
| Constipation | 9 (3.4) | 1 (0.4) |  | 2 (1.5) | 0 (0.0) | 0.255 | 1.000 |
| Fatigue | 47 (17.9) | 2 (0.8) |  | 6 (4.4) | 0 (0.0) | 0.000 | 0.548 |

NOTE：Data are presented as No. (%). Adverse events were graded according to the World Health Organization toxicity criteria.
